# Work-life conflict and associations with work- and nonwork-related factors and with physical and mental health outcomes: a nationally representative cross-sectional study in Switzerland

**DOI:** 10.1186/1471-2458-9-435

**Published:** 2009-11-30

**Authors:** Oliver Hämmig, Felix Gutzwiller, Georg Bauer

**Affiliations:** 1 Institute of Social and Preventive Medicine, University of Zurich, Hirschengraben 84, 8001 Zurich, Switzerland; 2 Center for Organizational and Occupational Sciences, Swiss Federal Institute of Technology Zurich, Kreuzplatz 5, 8032 Zurich, Switzerland

## Abstract

**Background:**

The aim of the present cross-sectional study was to examine work- and nonwork- related factors and physical and mental health outcomes associated with combined time- and strain-based work-life conflict (WLC) among adult employees living and working in Switzerland as well as possible gender differences in this regard.

**Methods:**

The data used for the study were taken from wave 6 of the nationally representative Swiss Household Panel (SHP) collected in 2004. The analysis was restricted to 4'371 employees aged 20 to 64 years. Trivariate crosstabulations and multivariate linear and logistic regression analyses stratified by gender were performed in order to calculate gender-specific prevalence rates (%), beta coefficients (β) and crude as well as multiple adjusted odds ratios (OR) as measures of association.

**Results:**

Every eighth person (12.5%) within the study population has a high or very high WLC score. Prevalence rates are clearly above average in men and women with higher education, in executive positions or managerial functions, in full-time jobs, with variable work schedules, regular overtime, long commuting time to work and job insecurity. Working overtime regularly, having variable work schedules and being in a management position are most strongly associated with WLC in men, whereas in women the level of employment is the strongest explanatory variable by far, followed by variable work schedules and high job status (managerial position). In both men and women, WLC is associated with several physical and mental health problems. Employees with high or very high WLC show a comparatively high relative risk of self-reported poor health, anxiety and depression, lack of energy and optimism, serious backache, headaches, sleep disorders and fatigue. While overall prevalence rate of (very) high WLC is higher in men than in women, associations between degrees of WLC and most health outcomes are stronger in women than in men.

**Conclusion:**

This important issue which up to now has been largely neglected in public health research needs to be addressed in future public health research and, if the findings are confirmed by subsequent (longitudinal) studies, to be considered in workplace health promotion and interventions in Switzerland as elsewhere.

## Background

The labour force participation of women in Switzerland has increased steadily, and is now one of the highest in Europe. In 1970, only 49% of all women in Switzerland aged 15 to 64 years were engaged in paid work, as opposed to 75% in 2007. For women aged 25 to 45 years with at least one pre-primary school-age child, the participation rate increased from 40% (1990) to 62% (2000) in a single decade. As a result, the number of working women (and men) with childcare and/or other private responsibilities and family obligations (e.g., elder care) is growing rapidly in Switzerland as in most other industrialised nations [1].

This cross-national trend has generated much scientific attention to the issue of reconciling work with private life under the rubric of work-family conflict (WFC) and a considerable body of research literature on the subject matter. Role conflicts between work and family life occur "when demands of participation in one domain are incompatible with demands of participation in the other domain" (p. 411) [2] or "when one's efforts to fulfil work role demands interfere with one's ability to fulfil family demands and vice versa" (p. 888) [3]. Researchers commonly distinguish between three forms of WFC (time-based, strain-based, and behaviour-based) [4,5] in two causal directions (work-to-family and family-to-work) [6-8]. However, most studies focus on time-based and strain-based work-to-family conflict.

Amidst a rapidly growing number of research studies on this topic, a new research tradition has developed centered on examining the causes and health- or work-related effects of WFC [9-14]. This literature has also enriched established research domains such as work-related health and stress research, by introducing the WFC construct as a risk factor for health or an explanatory factor for work stress [15-17]. Various studies revealed a number of health-related effects of WFC. Mental and physical health-related outcomes included increased substance abuse (especially problem drinking), greater psychological stress, more frequent depression and other mental disorders, burnout, and other psychosomatic symptoms including lack of appetite, sleep disorders, headaches or fatigue [3,15-24].

The research on WFC is commonly criticised for certain methodological and theoretical deficiencies [12,14,25-27]. Besides and partly in line with these qualified criticisms, the following limitations are addressed with the present study: First, the research has focused mostly on role conflicts between work and *family*. Thus, only a segment of the labour force is actually covered, namely those working men and women who have children or minors living at home. Singles, single parents, dual-income couples without children living at home, extended or patch-work families etc. are usually excluded from study populations in research on WFC.

Second, WFC research has been largely limited to certain subpopulations or professional categories--e.g., managers, self-employed, full-time workers, professors, teachers, students, dual-income couples, working parents/mothers--or certain settings--e.g., cities, schools, universities, public administration, hospitals, hotels, supermarkets. Mostly, the study populations are white-collar workers--i.e., well-educated members of the middle class employed by large companies. Unskilled employees and members of lower classes or ethnic/racial minorities have seldom been included in research efforts to date.

Third, most of the literature on WFC stems from English-speaking countries, in particular from North America. Due to differences in the work ethos, employment structures, and social norms related to work and family, the findings from North American studies cannot be transferred directly to the continental European context. To date, hardly any contributions from Switzerland and neighbouring countries with a scienticially sound basis have been published in this field.

In the present study, singles and childless dual career-couples are also included in the study population in order to overcome the traditionally limited scope of WFC research and its narrow focus on working men and women living with their own core families including dependent children. Hence, this study is focusing not only on role conflicts between work and *family*, but also considers role conflicts resulting from job demands that are difficult to bring in line with other role expectations and responsibilities in private life beyond family obligations. To document this broader scope and expanded study sample, we use the term "work-*life *conflict" (WLC).

Besides addressing the aforementioned shortcomings, the objectives or rather research interests of this study are:

• to analyse important work- and nonwork -related factors associated with WLC and to identify high prevalence groups in this regard;

• to study different mental and physical health correlates of WLC (as dependent variables); and

• to examine potential gender differences within these associations and between these correlates.

**Figure 1:**
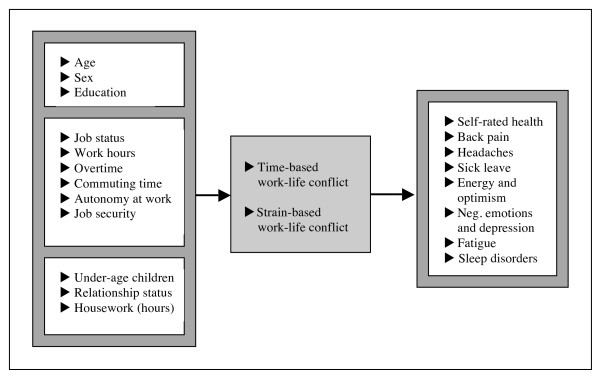
**Operationalised analytical model illustrating the factors and health outcomes that are expected to be associated with work-life conflict**.

The following graph illustrates the research interests of the present study and at the same time specifies the work- and nonwork related factors and the health outcomes that are expected to be associated with WLC and therefore included in the investigation (see Figure 1).

## Methods

### Data and study sample

To overcome one of the aforementioned limitations, the present study turns its attention basically to all the people that are engaged in paid work. Accordingly, the study sample also includes working men and women, singles and couples without minors living at home. Additionally, the study is not limited to specific and homogenous occupational groups or settings, but is based on a representative sample of the employed resident population in Switzerland including blue-collar workers. Cross-sectional data used for this study have been taken from the Swiss Household Panel (SHP). Launched in 1999, the SHP is a comprehensive telephone survey concerned with „Living in Switzerland“, carried out annually, and covering a broad range of topics including different work conditions and various aspects of health and well-being. It is based on a representative random sample of the entire resident population in Switzerland aged over 13. Between 1999 and 2004, the SHP lost more than 43% of its initial sample due to attrition. As dropouts increased over time, this loss was compensated by bolstering the original sample with new participants in 2004. Initially, this extended sample covers a total number of 5'375 households and 8'067 fully interviewed persons. Furthermore and in order to ensure the respresentativity of the sample, the data were weighted according to the last census in 2000 by gender, age, nationality, and region. Thus, weighted and unweighted data from the sixth data collection wave which includes the 2004 refreshment sample were used for this study. The study population was restricted to a subsample of 4'371 employed adults aged 20 to 64. Self-employed, retired or jobless persons as well as working people in adolescence and education, i.e. in secondary school or vocational training, are not comparable with employed adults aged 20 to 64 with respect to social roles and role pressures and conflicts and therefore were excluded from the study.

### Measures

#### Indicator variables

Since work-life balance (WLB) or imbalance in the SHP dataset had been measured originally by a single item--a dichotomous question on having major difficulties combining work and private life or not [28]--the following two questions were included in the SHP questionnaire in 2002 (wave 4) as measures of WLC on recommendation of the present authors:

• How strongly does your work interfere with your private life and family obligations more than you would like? (0 means „not at all“ and 10 „very strongly“)

• How difficult do you find it to disconnect from work (to leave the job behind) when the workday is over? (0 means „not difficult at all“ and 10 „extremely difficult“)

The two items were taken and adapted from the 18-item WFC-scale from Carlson et al. [4], i.e. reformulated according to the broader focus and scope of WLC and translated into German. They cover two of the three forms of role conflict, namely the time-based and the strain-based form. Given restrictions on the length of the questionnaire, the two items are intentionally limited to one causal direction, namely role conflicts that affect one's private life (work-to-life conflict) but not vice versa (life-to-work conflict).

#### Explanatory variables

In order to characterise potential groups at risk for WLC, demographic variables (age, sex, education) as well as work-related variables (job status, number of work hours, variable work schedule, overtime, commuting time, autonomy at work, and job security) were used. Job status was measured by a question about holding an executive or management position at work or not. Work hours were assessed with a dichotomous screening question about working part-time or full-time and a subsequent question that asked part-time workers to report the percentage of their part-time work. The reported level of employment was then transformed into work hours per week (part-time work of 50% and more equals 20 to 39 weekly work hours, part-time of less than 50% equals less than 20 weekly work hours). Variable work schedule was measured with a single question about having working hours that are either the same everyday or variable from day to day. To assess job security, the following question was used: "Would you say that your job is very secure, quite secure, a bit insecure or very insecure?". The only variable covered in the SHP that could be used as a measure for autonomy at work was the question:

„Within the responsibilities of your job, do you take part in decision-making, or provide advice on the management of the company?“. Furthermore, variables describing more or less demanding social roles and responsibilities in private life (parenthood or rather living with dependent children, time spent on housekeeping, relationship status) were used additionally as factors potentially associated with WLC. *Outcome variables: *General health status was measured by a single-item question on self-rated health, a 5-point Likert scaled item with response categories from 1 "very well" to 5 "very poor". This single-item global measure of general health status is a common health indicator in epidemiology and social science and well-established as a strong and independent predictor of general morbidity and mortality [29].

Physical health status was measured with two questions about currently suffering from bad back or lower back problems and from headaches or facial pains (scale from 1 „not at all“ to 3 „very much“). In addition, sick leave was measured by a question on how many days one has been affected by a health problem which made it impossible to carry out usual activity (work and housework). A declaration of 20 days or more over the last 12 months was considered long-term absence from work (or housework) due to health problems.

Mental health status was measured by four different variables assessing energy and optimism, negative emotions and depression, fatigue, and sleep disorders. Energy and optimism was measured by a question about having plenty of strength, energy and optimism (scale from 0 „never“ to 10 „always“). Negative emotions and depression were measured with a question about having feelings such as having the blues, being desperate, suffering from anxiety or depression (scale from 0 „never“ to 10 „always“). To measure fatigue, a question about suffering from general weakness, weariness, or lack of energy during the last 4 weeks was used (scale from 1 „not at all“ to 3 „very much“). Sleep disorders were measured by a similar question about suffering from difficulty in sleeping or insomnia (scale from 1 „not at all“ to 3 „very much“). Of course, some of the indicators like sick leave (or the number of days being absent from work) and sleep disorders possibly indicate both physical and mental health problems, but nevertheless were assigned to either one or the other health status. All of the health-related outcome variables were dichotomised for logistic regression analyses.

#### Adjusting variables

Beyond the already mentioned variables, some additional socio-demographic variables such as age, gender and education (as an indicator of socioeconomic status or social class) were used. These variables are most usually applied in multivariate statistical analyses to adjust or control for confounding or to disaggregate the data into different subgroups ("strata") in order to estimate group- or strata-specific measures of association, as done in the present study.

### Statistical analysis and construction of variables

A summary score ranging from 0 to 20 was created by summing the values of the two 11-point Likert scaled WLC-items with scores higher than 12 indicating comparatively high or very high degree of conflict (see Table 1). Cronbach's alpha for this 2-item scale was .53. As Likert scales (as the sum of several Likert items) are treated usually as interval data, especially when using more than five response categories and equal spacing (equidistancy) between the categories can be assumed, linear regression analysis can be conducted with the additive scale as the dependent variable to be explained.

**Table 1:** Indicators of time- and strain-based and aggregate work-life conflict (WLC) among 20-64 year old employees in Switzerland

Dimensions/indicators (categories)		Men		Women		Total	
				
	Score	N^1)^	%^2)^	N^1)^	%^2)^	N^1)^	%^2)^
Time-based WLC							
	0	367	19.2	605	28.0	972	23.3
*How strongly does your work interfere*	1-3	520	23.6	564	24.3	1084	23.9
*with your private life and family*	4-6	715	33.4	692	29.8	1407	31.7
*obligations more than you would like?*	7-10	481	23.8	411	17.9	892	21.0
							
Strain-based WLC							
	0	560	28.9	755	35.0	1315	31.8
*How difficult do you find it to*	1-3	688	31.7	631	27.1	1319	29.5
*disconnect from work when the*	4-6	532	25.2	572	24.6	1104	24.9
*work day is over?*	7-10	301	14.3	317	13.4	618	13.9
							
Level/degree of combined time- and							
strain-based WLC							
Inexistent	0	213	11.2	362	17.0	575	13.9
Very low	1-4	396	18.3	491	21.7	887	19.9
Low	5-8	675	32.1	674	29.4	1349	30.9
Moderate	9-12	510	24.5	476	20.9	986	22.8
High	13-16	256	12.6	222	9.3	478	11.0
Very high	17-20	29	1.3	43	1.7	72	1.5

^1) ^unweighted data; ^2) ^weighted data

In order to identify at risk groups, two-layered trivariate crosstabulations were conducted in order to compute prevalence rates of (very) high WLC for diverse population groups (differing in demographic as well as in work- and private life-related characteristics) and both sexes simultaneously. Subsequently, a multiple linear regression analysis was performed in order to investigate relevant independent work- and non work-related variables associated with WLC and to estimate partial standardized regression coefficients respectively beta coefficients (β) as measures of association. Finally, bivariate and multivariate logistic regression analyses with degree of WLC as the explanatory or independent variable and eight dichotomized physical and mental health indicators used as outcome measures or rather dependent variables were conducted in order to estimate odds ratios as approximate values for relative risks. For each of the binary coded outcome measures, two separate logistic regression models were fitted, one with crude odds ratios and without including covariates other than WLC (model 1) and the other showing odds ratios adjusted for age, education, job status and some additional work and private life characteristics as covariates and potential confounders (model 2). Results are presented as unadjusted odds ratios (OR) or adjusted odds ratios (aOR) with their 95% confidence intervals (95% CI). All regression analyses were carried out seperately for both genders. Cases excluded from the mentioned statistical analyses due to missing values are either very small in number or not due to refused answers. Most of all the missing values result from the exclusion of discrete response categories (e.g., 'don't know', 'not applicable') from ordinally scaled variables. Therefore, missing values in the present study are expected to be randomly distributed within the study population and not cause systematic bias or error due to self-selection. Thus, missing values are not assumed to influence the findings.

## Results

### Prevalence of (very) high WLC in different subpopulations

Every eighth person of the study population (12.5%) has a high or very high work-life imbalance or conflict, as measured by the two indicators of time- and strain-based WLC (see Table 1). Another fourth (22.8%) of the sample shows moderate work-life (im)balance. Overall, more men (13.9%) report high or very high WLC than women (11%). But if women and men with the same job status and level of employment are compared, women in management positions (20.2%) and full-time jobs (15.1%) show higher prevalences of (very) high WLC than their male counterparts in the same positions (18.9%) and jobs (14.3%) (see Table 2).

**Table 2:** Proportion of high or very high work-life conflict in different subgroups of the employed population aged 20-64 years in Switzerland

	Men		Women	
		
	%^1)^	p-value	%^1)^	p-value
**Total study population**	**13.9**		**11.0**	
*Demographics*				
Age				
20-29 years	8.8	< .001	12.4	≤ .05
30-39 years	15.3		10.7	
40-49 years	16.7		10.8	
50-64 years	12.8		10.4	
Education (highest level of education achieved)				
(Incomplete) compulsory schooling	12.1	<. 001	8.5	<. 001
Apprenticeship, general training	10.7		9.1	
Secondary (vocational, technical, matura)	16.8		11.5	
University	17.0		17.1	
*Work life characteristics*				
Management position (high job status)				
yes	18.9	< .001	20.2	< .001
no	10.1		8.5	
Work hours (hours of paid work per week)				
< 20 hours (part-time job)	2.7	< .001	5.4	< .001
20-39 hours (part-time job)	14.5		11.6	
≥ 40 hours (full-time job)	14.3		15.1	
Variable work schedule				
yes	15.4	< .001	12.7	< .001
no	11.9		9.2	
Regular overtime				
yes	17.3	< .001	15.7	< .001
no	8.8		8.3	
Commuting time (one-way)				
up to 30 minutes	13.0	< .001	10.6	< .001
31-60 minutes	12.5		11.2	
more than 60 minutes	18.2		14.0	
Participation in decision-making (autonomy at work)				
yes	16.0	< .001	12.1	< .01
no	8.7		9.4	
Job security				
Very/quite secure job	13.0	< .001	10.3	≤ .05
A bit/very insecure job	19.7		15.2	
*Private life characteristics*				
Living with dependent children				
yes	17.4	< .001	10.5	> .05
no	11.4		11.2	
Having a long-term relationship				
yes	14.6	< .01	10.9	> .05
no	9.9		11.2	
Housework/housekeeping (hours per week)				
≤ 5 hours per week	15.6	≤ .05	10.8	< .001
6-20 hours per week	11.1		12.5	
> 20 hours per week	10.3		8.2	

Number of cases^2)^	2079		2268	

^1) ^weighted data; ^2) ^unweighted data

In general, men and women with higher education, in executive or managerial positions, in full-time jobs, with variable work schedules, regular overtime, long commutes (more than 60 minutes each way), with self-perceived job insecurity, and who participate in decision-making at work show higher than average rates of high or very high WLC (see Table 2). This applies also to employed men with dependent children living at home and in a long-term relationship. Against expectations and among both men and women, more housekeeping hours do not increase the prevalence of (very) high WLC.

### Associations between work- and nonwork -related factors and WLC

Multiple linear regression analyses show that most of the work and private life characteristics used and presented in Table 2 are significantly and positively associated with WLC even when adjusting for all other variables (see Table 3). But gender differences can be observed. Regular overtime work, having a variable work schedule and being in a management position are most strongly associated with WLC among men, whereas among women, the number of work hours per week as an indicator of the level of employment and workload is the strongest explanatory variable by far (β = .28), followed by a variable work schedule and high job status (management position). For both genders, having an insecure job, long commutes, and living with dependent children are also significantly associated with WLB. On the contrary, decision-making at work, relationship status and time spent on housekeeping are not associated at all with WLC for either men or women. Gender itself is a relevant control variable in the consolidated regression model, as being female is clearly associated with higher degree of WLC (β = .11).

**Table 3:** Work- and nonwork-related factors that are associated with work-life conflict among 20-64 year old employees in Switzerland

*Dependent variable*:	Men	Women	Total
Work-life conflict (0-20)			
	*β*	*β*	*β*
*Independent variables*:			
Management position (dummy)	.11***	.09***	.11***
Work hours (per week)	.06**	.28***	.21***
Variable work schedule (dummy)	.12***	.09***	.10***
Regular overtime (dummy)	.14***	.08***	.11***
Commuting time (minutes per day each way)	.08***	.06*	.07***
Participation in decision-making (dummy)	n.s.	n.s.	n.s.
Job insecurity (dummy)	.08***	.07***	.08***
Living with dependent children (dummy)	.09***	.08**	.07***
Not being in a long-term relationship (dummy)	n.s.	n.s.	n.s.
Housework/housekeeping (hours per week)	n.s.	n.s.	n.s.
*Control variables*:			
Sex (female)	-	-	.11***
Age	n.s.	n.s.	n.s.
Education (highest level achieved)	.07**	.10***	.09***

Adjusted R-Squared	.111	.135	.126

Number of cases in model	1863	1920	3784

*p ≤ .05; **p < .01; ***p < .001; n.s. = not significant (p > .05)

### Physical and mental health correlates of WLC

Employees with high or very high WLC demonstrate a significantly higher relative risk of poor well-being and physical and mental health problems compared to those with very low or no WLC at all (see Tables 4 &5). Strong associations between WLC and health outcomes under study with clear gradient, i.e. steadily increasing OR with cumulative degree of WLC, were observed almost across the board which suggest causality (model 1). These findings are consistent and even magnified when adjusting for age, educational level and different work and private life characteristics which may be associated with both WLC (see Table 3) and health, suggesting possible confounding (model 2).

**Table 4:** Work-life conflict as a potential risk factor for general and physical health among 20-64 year old employees in Switzerland

	Fair to very poor self-rated health				Suffer a lot from backache or lower back pain			
		
	Men (6.9%)		Women (10.6%)		Men (8.6%)		Women (14.2%)	
				
	OR	95%-CI	OR	95%-CI	OR	95%-CI	OR	95%-CI
**Model 1^†^**		n = 2079		n = 2268		n = 2078		n = 2268
Work-life conflict								
very low (0-4)	1		1		1		1	
low (5-8)	1.24	0.78-1.95	0.99	0.69-1.44	0.67	0.44-1.00	1.00	0.73-1.37
moderate (9-12)	1.09	0.66-1.81	**1.86**	1.31-2.66	0.71	0.46-1.09	1.26	0.90-1.75
(very) high (13-20)	1.77	1.05-3.00	1.27	0.80-2.02	1.23	0.78-1.93	1.54	1.05-2.26
								
**Model 2^††^**		n = 2034		n = 2180		n = 2033		n = 2180
Work-life conflict								
very low (0-4)	1		1		1		1	
low (5-8)	1.57	0.96-2.55	1.12	0.76-1.64	0.75	0.50-1.14	1.05	0.76-1.46
moderate (9-12)	1.58	0.93-2.70	**2.15**	1.47-3.14	0.87	0.56-1.36	1.40	0.99-1.99
(very) high (13-20)	**2.67**	1.50-4.76	1.49	0.90-2.47	1.66	1.03-2.67	1.78	1.17-2.69

								

	**Suffer a lot from headaches or facial pain**				**Sick leave/long-term absence from work**			
		
	**Men** **(4.5%)**		**Women** **(13.1%)**		**Men** **(7.9%)**		**Women** **(8.9%)**	
				
	**OR**	**95%-CI**	**OR**	**95%-CI**	**OR**	**95%-CI**	**OR**	**95%-CI**

**Model 1^†^**		n = 2079		n = 2267		n = 2073		n = 2248
Work-life conflict								
very low (0-4)	1		1		1		1	
low (5-8)	1.51	0.82-2.78	1.28	0.91-1.80	0.77	0.52-1.15	0.96	0.65-1.40
moderate (9-12)	1.64	0.87-3.11	**1.90**	1.35-2.69	0.69	0.45-1.08	1.19	0.79-1.77
(very) high (13-20)	**2.77**	1.44-5.36	**2.31**	1.56-3.44	0.57	0.32-1.02	1.37	0.85-2.19
								
**Model 2^††^**		n = 2034		n = 2179		n = 2028		n = 2160
Work-life conflict								
very low (0-4)	1		1		1		1	
low (5-8)	1.70	0.90-3.21	1.25	0.87-1.79	0.95	0.63-1.43	1.03	0.69-1.55
moderate (9-12)	1.94	0.99-3.77	**1.89**	1.31-2.74	0.92	0.58-1.46	1.32	0.86-2.03
(very) high (13-20)	**3.20**	1.58-6.48	**2.48**	1.62-3.81	0.79	0.44-1.44	1.52	0.91-2.54

^† ^Unadjusted odds ratios (OR); bold figures = highly significant OR (p < .01)

^†† ^Odds ratios adjusted (aOR) for age, educational level, job status, work hours per week, job insecurity and living with dependent children; bold figures = highly significant aOR (p < .01)

**Table 5:** Work-life conflict as a potential risk factor for mental health among 20-64 year old employees in Switzerland

	Have seldom or never plenty of strength, energy and optimism (0-4)				Have often or always negative emotions and depression (6-10)			
		
	Men (4.8%)		Women (5.6%)		Men (4.4%)		Women (8.3%)	
				
	OR	95%-CI	OR	95%-CI	OR	95%-CI	OR	95%-CI
**Model 1^†^**		n = 2075		n = 2262		n = 2078		n = 2266
Work-life conflict								
very low (0-4)	1		1		1		1	
low (5-8)	0.83	0.47-1.45	1.56	0.95-2.55	0.94	0.51-1.73	1.32	0.84-2.06
moderate (9-12)	1.06	0.60-1.88	1.91	1.14-3.20	1.33	0.73-2.42	**2.71**	1.77-4.13
(very) high (13-20)	1.33	0.70-2.52	1.77	0.95-3.30	2.23	1.20-4.15	**3.19**	1.99-5.12
								
**Model 2^††^**		n = 2030		n = 2174		n = 2033		n = 2178
Work-life conflict								
very low (0-4)	1		1		1		1	
low (5-8)	0.94	0.52-1.69	1.56	0.92-2.63	1.11	0.60-2.06	1.47	0.92-2.34
moderate (9-12)	1.28	0.71-2.32	2.01	1.16-3.47	1.66	0.89-3.08	**3.12**	1.99-4.88
(very) high (13-20)	1.84	0.94-3.60	1.82	0.93-3.53	**3.16**	1.63-6.11	**3.84**	2.30-6.42

								

	**Have a lot of difficulty in sleeping or insomnia**				**Suffer a lot from general weakness, weariness, lack of energy**			
		
	**Men** **(4.1%)**		**Women** **(9.7%)**		**Men** **(4.5%)**		**Women** **(13.5%)**	
				
	**OR**	**95%-CI**	**OR**	**95%-CI**	**OR**	**95%-CI**	**OR**	**95%-CI**

**Model 1^†^**		n = 2079		n = 2268		n = 2078		n = 2266
Work-life conflict								
very low (0-4)	1		1		1		1	
low (5-8)	1.05	0.56-2.00	1.59	1.06-2.38	1.45	0.81-2.60	1.32	0.93-1.87
moderate (9-12)	1.41	0.74-2.68	**1.97**	1.29-2.99	1.80	1.00-3.27	**2.41**	1.71-3.39
(very) high (13-20)	**3.16**	1.69-5.89	**3.59**	2.32-5.55	2.34	1.23-4.46	**3.67**	2.52-5.34
								
**Model 2^††^**		n = 2034		n = 2180		n = 2033		n = 2178
Work-life conflict								
very low (0-4)	1		1		1		1	
low (5-8)	1.21	0.63-2.31	**1.87**	1.23-2.85	1.80	0.98-3.30	1.39	0.96-2.01
moderate (9-12)	1.74	0.90-3.35	**2.39**	1.53-3.73	2.23	1.19-4.17	**2.57**	1.78-3.70
(very) high (13-20)	**4.14**	2.14-8.01	**4.67**	2.90-7.53	**3.38**	1.70-6.70	**3.77**	2.50-5.69

^† ^Unadjusted odds ratios (OR); bold figures = highly significant OR (p < .01)

^†† ^Odds ratios adjusted (aOR) for age, educational level, job status, work hours per week, job insecurity and living with dependent children; bold figures = highly significant aOR (p < .01)

Concerning general health status and physical health problems (see Table 4), both men and women with high or very high WLC (most „exposed“ group) showed an increased risk of reporting only moderate or poor health (men: aOR = 2.7 vs. women: aOR = 1.5), suffering a lot from backache or lower back pain (aOR = 1.7 vs. 1.8) and from headaches (aOR = 3.2 vs. 2.5) compared to their counterparts with very low WLC (reference group). But no significant association was observed between the degree of WLC and the occurrence of long-term absence from work (sick leave) among either men or women.

A high or very high level of WLC was associated with mental health problems, even more strongly than with physical health problems (see Table 5). This applies basically to both men and women, even though associations are slightly stronger in women than in men. Among men with one exception, only the „most exposed“ group (high or very high WLC) shows a significantly increased risk of mental health problems--i.e., lack of energy and optimism, negative feelings and depression, sleep disorders, and fatigue--compared to the "least exposed" reference group (very low WLC), whereas among women, already a moderate level of WLC was associated with a significantly increased risk of mental health problems. As for physical health problems, adjusting for age, education and different private life characteristics and work-related variables (model 2) did not make much difference in the results but rather increased the strength of the association. Adjusted OR range from 1.8 to 4.7. And again, a near consistent gradient was found in general and among both men and women: the higher the level of WLC, the higher the relative risk of negative emotions and depression, sleep disorders, and fatigue (or general weakness and weariness).

## Discussion

The present study sought to answer explicit research questions about groups with elevated levels of WLC, important correlates and health-related outcomes of WLC among adult employees in Switzerland as well as potential gender differences in this regard.

Regarding the first research question about identifying groups at risk of experiencing WLC and work-related and other factors associated with WLC, the study clearly showed that high numbers of hours spent at work, regular overtime, long commutes and a high job status (management position) are all associated with elevated levels of WLC. These findings make sense, since high work demands compete with other social roles and private obligations for limited time, energy and psychological resources, thereby leading to multiple burden and increasing role conflict between work and private life [30-33]. Other significant and relevant influencing factors found in the present study were job insecurity and living with dependent children. Interestingly, a differentiated composite score for WLC composed of 2 items yielded more or less the same risk groups with above average prevalence rates as a dichotomous single-item measure (yes/no question) about one's difficulties in reconciling work with private life [28].

Nearly all of the above-mentioned results are fully in line with international research showing that high job involvement and workload and in particular a large time commitment to work as well as job insecurity and childcare responsibilities are antecedents of WFC [2,7,10,20,34]. On the other hand, three of the findings go against expectations and/or are not consistent with international WFC research. Firstly, contrary to international studies showing an association between flexible work schedules (such as flextime) and lower WFC [10,34-36], in the present study variable work schedule in terms of alternating or changing working hours from day to day was found to be associated with higher WLC compared to fixed, constant working hours. Variable working hours in this sense may not represent real self-determined time flexibility at work, but poorly predictable and other-directed working hours which of course are not a resource for balancing or reconciling one's work with his/her private life.

Secondly, in international studies, job autonomy--also known as influence at work, decision latitude, operational flexibility or work-time control--was found to be related to positive spillover between work and family and to be protective with regard to WFC [31,35,37-39]. But in the present study, job autonomy was not significantly associated with WLC. This may be due to a measurement problem. Participation in decision-making at work as assessed in the SHP survey (see section on "Measures") may not imply real autonomy in how and when the job gets done and is therefore not beneficial for one's WLB.

Thirdly, housekeeping has also been shown to be another antecedent of WFC in international studies [20], but that finding was not replicated in the present study. Since the level of engagement in paid work varies depending on the time spent on housework in our study, more housekeeping hours per week often go along with lower level of employment and a decreased number of weekly work hours which have been proven to be beneficial with regard to WLB.

In spite of the oft-quoted dual burden of working women and despite the fact that women mostly spend more hours in combined work and family activities and have a greater total load than men [8,40], in the present study women at large were found to be somewhat less affected by WLC than men, as reported in other studies [20,41]. Every seventh man, but only every ninth woman in the study population showed a (very) high WLC. This finding is in line with gender role theory and perspective and the so-called domain salience hypothesis postulating that role pressures and conflict are itensified when either work or family roles are salient and central to the person's self-concept [5]. In other words: the more important a role is to an individual, the more time and energy that individual will invest in it and the more likely are conflicts with other roles [42]. In light of still persisting traditional gender roles [8] it is assumed that the work domain is a greater source of role pressures and conflict for men while the family domain is a greater source of role pressures and conflict for women [40,43]. Based on this argumentation and according to the rational view of gender differences [8], it has been concluded that men report more work-to-family/life conflict than women and women report more family/life-to-work conflict than men [44].

But in fact, the gender difference found in this study and going in the assumed direction is caused by an under-representation of women in full-time jobs and management positions in the study population just as well as in the general (working) population. Only 34.2% of women in the study sample work full-time and just 21.1% are in a management position, whereas 86.9% of all men work full-time and 42.8% are in a leading position. If prevalence rates are adjusted for job status and level of employment--i.e., if women and men working full-time and in the same job position are compared (see Table 2) --the gender difference with respect to WLC decreases, disappears entirely or is even reversed.

Findings in the research literatur on WFC referring to gender differences are contradictory [44]. Some research has found no gender differences [40,42,45], whereas other studies have reported gender differences in this regard mostly in the sense and direction of women experiencing more WFC than men [8,36].

Beyond this, some interesting gender differences were found. While in men regular overtime, variable work schedule, and high job status (executive or management position) were most strongly associated with a higher level of WLC, in women the time committed to work or rather the number of contracted hours spent at work was by far the strongest explanatory variable. Job insecurity, long commutes, and living with dependent children in contrast turned out to be additional factors of equal strength among both men and women. These findings need to be replicated by others before they can be generalised, since they are inconsistent with findings from other studies like Jansen et al.'s cohort study [20] that found job insecurity to be an antecedent of WFC especially for men, and overtime work, long commuting time and having dependent children to be greater risk factors for women than for men.

Altogether the variance explained by all explanatory and controlling variables included in the linear regression model was only 11.1% for men and 13.5% for women. This large 'unexplained variance' suggests that there are other relevant, but unconsidered factors especially in the nonwork domain such as family obligations beyond childcare, leisure activities not covered in the SHP, and personality traits or individual preferences concerning the need for WLB, work ethos, commitment to family etc.

As far as the second research question regarding health correlates of WLC is concerned, findings of the present study confirmed that WLC is quite strongly associated with impaired general well-being, reduced physical health and limited mental health. These findings concur with international studies. Allen and Armstrong [46], Grandey and Cropanzano [33] and Frone et al. [24] found that WFC was associated with poor physical health. Others showed that WFC had an adverse affect on mental health [19,47] or was strongly related to depression [24], fatigue [20] and psychiatric disorders [3].

Results show reasonably strong associations with a clear gradient (odds ratios increase in tandem with WLC) almost throughout, and associations are not diminished when adjusting for age, education and different work and private life characteristics. Those men and women who report a moderate or (very) high WLC show almost consistently (although not always significantly) an increased relative risk for general ill-being and different mental and physical health problems (e.g., poor self-rated health, serious headaches, negative emotions and depression, sleep disorders, fatigue) in comparison with those with inexistent or very low WLC (reference group). Multiple adjusted odds ratios for the most exposed group with high or very high WLC range from 1.5 to 4.7 depending on the health outcome. Only sick leave or rather being absent from work for 20 days or more in the past 12 months constitutes an exception to this pattern and is not associated with WLC at all.

Interestingly, when looking at mental health problems as outcome variables, women show slightly higher odds ratios--i.e., somewhat stronger associations between WLC and mental health impairments--than men. This finding is supported by a previous longitudinal study of Kinnunen et al. [45] who found work-to-family conflict to be more detrimental to women's satisfaction and well-being than that of men. An explanation for this gender difference might be that negative spillover or interference from work to family/private life is more stressful and problematic mentally for women because the family role and private life domain is more important to the woman's self-concept and social identity [5,8,40]. Men in turn obtain their personal and social resources (e.g., self-esteem, social status, social identity, social support) more from work and are therefore less affected mentally by such role conflicts from work to family or private life and by negative sanctions as a result of noncompliance with family role demands [5,8,40]. So work-to-life conflict as measured in the present study may have more adverse effects on women's (mental) health and well-being, whereas life-to-work conflict which was not assessed in the study may impair men's health.

### Strengths and limitations of the present study

One of the goals of the present cross-sectional study was to overcome some of the major limitations of the international research on WFC. With their generally homogenous, non-representative samples, findings between studies in this field of research usually cannot be compared with each other or transferred to other groups, much less the general population or the entire labor force. With very few exceptions, there are practically no representative population-based studies. By broadening the limited scope and traditionally narrow focus on WFC, by using nationally representative survey data, and by having a study population that includes not only white-collar employees or specific subpopulations or occupational categories but also blue-collar workers, findings from the present study can be generalised to the entire employed population in Switzerland, thereby overcoming the widespread middle-class bias in this field of research and partly compensating for the lack of evidence in this country.

Of course, the study also has some methodological limitations. WFC research has been criticized for an overreliance on cross-sectional study designs [25], and this criticism applies just as well to the present study which does not permit causal inferences. Since WLC and health outcomes have been measured simultaneously, causality is uncertain and doubtful. Statistical associations found in observational studies can never prove causal relationships [48]. Cross-sectional data in particular cannot respond to the question if exposure precedes the outcome - a key criterion on Austin Bradford Hill's widely-cited list of criteria to be considered before inferring causation when observing a statistical association [49]. And although longitudinal data by contrast comply with this criterion of temporality, longitudinal data and evidence are not sufficient to fully allow the assumption of causality [45] and to conclude from association to causation either. None of Hill's criteria are sufficient and none, perhaps with the exception of temporality, are absolute conditions and sine qua non for causation [50].

Yet strong associations with a clear gradient (linearly rising relative health risks with increasing WLC) found consistently between WLC and diverse (mental) health outcomes suggest a potential cause-effect relationship according to two additional criteria of causation, namely strength and linearity (gradient) [49]. And in addition, considering alternative explanations by stratification of analysis and/or controlling statistically for potentially confounding factors is a useful strategy to distinguish effects of exposure from those of confounding and another way to "study association before we cry causation" [49].

However not only the question of causality remains open-ended in this study, but even the direction of causation is unclear since in recent years researchers found evidence and support for reverse causation between WFC and health. Several longitudinal studies on antecedents and consequences of WFC have shown lagged effects indicating the hypothesised causal relationship [51,52] as well as reversed effects and bidirectional or reciprocal relations [51,53-56] between work-family interaction or work-home interference on the one hand and different work stressors or health outcomes on the other.

Another point of criticism in most WFC research is poor measurement [25]. This is also a major limitation of the present study. By relying on secondary analysis of existing data, we were strongly limited in the measurement of WLC as a multidimensional construct. WLC was assessed with a 2-item scale containing just two out of three distinguished forms (time- and strain-based) aligned to one causal direction (work-to-life conflict) only and showing a low reliability coefficient (alpha = .53). The third form (behavior-based) and the other type or direction of conflict (life-to-work conflict) were not measured at all in the SHP and therefore could not been used for this study. In other words, assessment of WLC in the present study is far away from the well-established and best-validated multiple-item measures such as the 18-item scale of Carlson et al. [4] or the 12-item scale of Netemeyer et al. [57].

Since health and social sciences increasingly use multiple-item scales to measure complex multidimensional constructs such as self-esteem, health status, stress, job satisfaction, and many others, there is an ongoing debate on the validity and reliability of single-item measures compared to multiple-item measures [58,59]. However, by replacing a dichotomous single-item measure of WLC [28] with a score composed of two 11-point Likert-scaled items, there are fewer concerns at least about validity and potential measurement problems in this study. Furthermore, the construct validity of the scale used is bolstered by finding similar groups with elevated levels of WLC and mostly consistent associations with different health outcomes in other research on WFC.

However, poor measures (especially when measured by questionnaire) can produce information bias and as a result misclassification bias. Misclassification of exposure which is independent of other (measurement) errors and non-differential with respect to the outcome results in estimation bias towards the null value (which is 1 when using common measures of association such as the odds ratio) [60,61]. When exposure is uncommon or when misclassification of exposure and outcome is non-differential but not independent of one another, bias away from the null can result [62,63].

In the present study, exposure is not rare, but using single-source self-report survey data carries a potential risk of non-differential non-independent misclassification of exposure and outcome which occurs when misclassification errors are correlated. This is the case when both work-life conflict (exposure) and physical and mental health disorders (outcomes) are either overreported or underreported by the same subjects leading to an overestimation or an underestimation of the true association. 'Finding' a non-existing association due to (dependent) misclassification would be a major problem.

Using different sources of information or data for exposure and outcome would have strongly reduced the risk of dependent misclassification [61,63] and of common method variance or bias. In the present study, using a differentiated ordinal scale instead of a dichotomous variable as a measure of exposure (which is by nature gradual and dose-dependent and not a binary state) may have reduced the risk of information and misclassification bias. Similar associations found consistently for all health outcomes may indicate that the probability of dependent misclassification is rather low. It is not plausible otherwise that the same measurement or misclassification error could have been observed for all health outcomes.

## Conclusion

In conclusion, the results from the present study provide additional evidence for certain and basically changeable working conditions and private life situations as relevant factors associated with WLC and, most important, for different negative health outcomes as correlates of WLC. The findings complement findings from our own previous research [28] filling further the still existing research gap and lack of evidence in Switzerland referring to this. Concerning the main findings, the study results are largely consistent with the risk factors and health effects found in international studies and documented in the research literature on WFC. This could not be assumed since the present study differs significantly from most international studies on this topic with respect to the study population. We studied a large cross-section of the general working or rather employed population in Switzerland including childless singles or couples and blue-collar workers, whereas most international studies are based on small, specific and homogenous samples of 'middle class', white-collar workers excluding childless singles or couples a priori.

With more than 12% of the employed population of Switzerland being affected by relatively high or very high WLC and the high relative risk of poor self-rated health and various physical and mental health problems found among the "most exposed" group, it can be said that WLC or work-life imbalance is an issue of great importance and relevance for the public's health in Switzerland. WLC was clearly associated with both physical and mental health problems and general ill-being. To date and with the exception of very few studies (e.g. Fuss et al. [64] or Sandmark [65]), public health and social epidemiology, as one of its major scientific disciplines, have not taken up the issue of work-life conflict or (im)balance much less considered its role as potential health determinant. Research on health-related effects of WFC traditionally has been and remains a domain of (occupational) health psychologists. Hopefully, our findings will help place WLC on the research agenda of public health in the future.

### Directions for future research and practical implications

Since there is very little or no research at all on WLC done and/or published in Central European countries and especially in Switzerland, more empirical evidence and research on this topic is needed. An issue for future research is to study possibly different health effects of the three forms and two directions of work-life conflict. New data measuring all six dimensions, the three forms (strain-based, time-based, behaviour-based) as well as the two directions (work-to-life and life-to-work conflict), of the WLC construct should be collected or additional items should be added to the SHP to make this possible. Beyond that, potential positive spillover effects on health (and other aspects) resulting from the interaction or integration of the two life domains are still largely unexplored, not only in Switzerland. And last but not least, future research in Switzerland should take advantage of the longitudinal design of the SHP in order to illuminate causal pathways and relationships between WLC and health. In recent years WFC was considered both cause and consequence of ill-health as different longitudinal studies have found support for reciprocal relations or reversed causality [28].

Similar studies and consistent findings for Switzerland based on longitudinal data would have practical implications for devising preventive or health promoting interventions at the workplace and identifying possible points of entry to break the potential downward spiral and vicious circle between WLC and ill-health.

## Competing interests

The authors declare that they have no competing interests.

## Authors' contributions

OH conceived the study, drafted the manuscript and performed the statistical analysis. FG has been involved in revising the manuscript critically with regard to content and statistical approach. GB has contributed considerably to the conception of the study and participated in the interpretation of the results. All authors have read the manuscript repeatedly and approved its final version.

## Pre-publication history

The pre-publication history for this paper can be accessed here:

http://www.biomedcentral.com/1471-2458/9/435/prepub
